# Inhibition of miR-21 restores RANKL/OPG ratio in multiple myeloma-derived bone marrow stromal cells and impairs the resorbing activity of mature osteoclasts

**DOI:** 10.18632/oncotarget.4398

**Published:** 2015-06-24

**Authors:** Maria Rita Pitari, Marco Rossi, Nicola Amodio, Cirino Botta, Eugenio Morelli, Cinzia Federico, Annamaria Gullà, Daniele Caracciolo, Maria Teresa Di Martino, Mariamena Arbitrio, Antonio Giordano, Pierosandro Tagliaferri, Pierfrancesco Tassone

**Affiliations:** ^1^ Department of Experimental and Clinical Medicine and T. Campanella Cancer Center, Magna Graecia University, S. Venuta University Campus, Catanzaro, Italy; ^2^ ISN-CNR, Roccelletta di Borgia, Catanzaro, Italy; ^3^ Department of Human Pathology and Oncology, University of Siena, Siena, Italy; ^4^ Sbarro Institute for Cancer Research and Molecular Medicine, Center for Biotechnology, College of Science and Technology, Temple University, Philadelphia, PA, USA

**Keywords:** miR-21, miRNAs, multiple myeloma bone disease, OPG, RANKL

## Abstract

miR-21 is an oncogenic microRNA (miRNA) with an emerging role as therapeutic target in human malignancies, including multiple myeloma (MM). Here we investigated whether miR-21 is involved in MM-related bone disease (BD). We found that miR-21 expression is dramatically enhanced, while osteoprotegerin (OPG) is strongly reduced, in bone marrow stromal cells (BMSCs) adherent to MM cells. On this basis, we validated the 3′UTR of OPG mRNA as miR-21 target. Constitutive miR-21 inhibition in lentiviral-transduced BMSCs adherent to MM cells restored OPG expression and secretion. Interestingly, miR-21 inhibition reduced RANKL production by BMSCs. Overexpression of protein inhibitor of activated STAT3 (PIAS3), which is a direct and validated target of miR-21, antagonized STAT3-mediated RANKL gene activation. Finally, we demonstrate that constitutive expression of miR-21 inhibitors in BMSCs restores RANKL/OPG balance and dramatically impairs the resorbing activity of mature osteoclasts. Taken together, our data provide proof-of-concept that miR-21 overexpression within MM-microenviroment plays a crucial role in bone resorption/apposition balance, supporting the design of innovative miR-21 inhibition-based strategies for MM-related BD.

## INTRODUCTION

Multiple myeloma (MM) is a malignancy characterized by accumulation of monoclonal plasma cells (PCs) within the bone marrow (BM). The direct interplay between MM cells and BM cell components has a crucial role in disease pathogenesis and skeletal destruction, a cardinal clinical feature of MM. Adhesion of malignant PCs to BM stromal cells (BMSCs) supports MM cell proliferation and survival, osteoclast (OCL)-dependent resorptive activity and inhibits osteoblast (OBL)-dependent bone formation, affecting skeletal homeostasis [1]. Specifically, BMSCs interact with MM cells and secrete high levels of interleukin-6 (IL-6), a major MM cell growth factor, osteoclast-activating cytokines such as IL-1β, tumor necrosis factor-α (TNFα), and the receptor activator of nuclear factor κB-ligand (RANKL), a key factor and dominant mediator of osteoclast differentiation, activation and survival [2, 3]. Moreover, MM cells inhibit osteoprotegerin (OPG) secretion by BMSCs and OBLs, thereby inducing severe imbalance in RANKL/OPG ratio, which is the main driver of bone homeostasis [4]. OPG belongs to TNF receptor ligand superfamily and has been identified as critical player in normal bone turnover. In fact, OPG is a decoy receptor of RANKL: it antagonizes RANKL binding to RANK and consequently preserves the integrity of bone mass [5]. The biological relevance of this system is demonstrated by the development of severe osteoporosis and osteopetrosis in OPG- and RANKL- knockout mice, respectively [6, 7]. A balanced RANKL/OPG ratio is therefore essential for physiologic bone-remodeling. Although it is well known that MM cells induce excessive osteoclastic-mediated bone resorption, the biologic mechanisms underlying MM-induced OPG downregulation remain to be elucidated. Currently, the clinical management of MM-related bone disease (BD) is mainly based on the use of bone modifying agents (BMAs), such as bisphosphonates [8–14], that promote OCL apoptosis. Other BMAs of clinical relevance are Denosumab, a monoclonal IgG2 antibody raised against RANKL [15], and Dasatinib, that suppresses OCL formation by inhibiting c-Fms on OCL progenitors [16]. Additionally, anti-MM agents targeting the BM microenviroment, such as bortezomib, that decreases RANKL in MM patients serum, or lenalidomide, that overcomes cytokine and BMSCs-mediated drug-resistance, are considered active agents against MM-related BD. Despite currently available treatments, progressive skeletal destruction still remains a relevant clinical problem for MM patients. Therefore, a better understanding of the molecular networks involved in sustaining MM-related BD is eagerly awaited to design novel therapeutic strategies. The recent advances in microRNAs (miRNAs) biology have disclosed a new exciting scenario for the design of innovative therapeutics for MM and its related BD [17–22]. miRNAs are a class of small, non-coding RNAs of 19 to 25 nucleotides, that act as negative regulators of gene expression at the post-transcriptional level by binding to the 3′ untranslated region (3′UTR) of their target mRNAs, thereby leading to its degradation or translation repression. miRNAs play a pivotal role in key events regulating cell differentiation, proliferation, metabolism and apoptosis, and their aberrant activity is involved in the pathogenesis of human cancer [23]. So far, some relevant miRNAs have been found dysregulated in MM [17–20, 24]: miR-34a [25, 26], −29b [27–29], −199a–5p [30], −30–5p [31], −15a/16–1 cluster [32] and −125b–5p [33] act as tumor suppressor miRNAs, whereas miR-21 [34], −221/222 [35, 36], −125a [37] and −17/92 cluster [38], are considered onco-miRNAs. Some of these miRNAs have also been found to be involved in MM-related BD. Indeed, miR-29b inhibits OCL differentiation and activity, therefore abrogating MM-mediated bone resorption [27, 39], while promotes osteoblastogenesis and the mineralization phase [40]. Moreover, among miRNAs involved in MM, it has been previously demonstrated that aberrant expression of miR-21 has high biologic relevance since it is regulated by IL-6 through STAT3-pathway activation [41]. Indeed, miR-21 is upregulated in OCLs [42] and BM mononuclear cells of MM patients [43], suggesting a crucial role for miR-21 within the BM *milieu* [34]. Moreover, high levels of miR-21 prevent MM cells apoptosis triggered by dexamethasone, doxorubicin, or bortezomib, while its downregulation rescues sensitivity to these agents, suggesting also its relevant role as modulator of drug-resistance [44]. In this light, we investigated whether miR-21 may play a role in the complex network sustaining the MM-related BD. Indeed, findings presented here provide proof-of-principle that miR-21 has a pivotal role in OPG downmodulation and RANKL upregulation, disclosing a relevant area of investigation for the design of novel therapeutic strategies against MM-related BD.

## RESULTS

### Adhesion to MM cells upregulates miR-21 and downregulates OPG in HS-5 BM stromal cells

**Figure 1:**
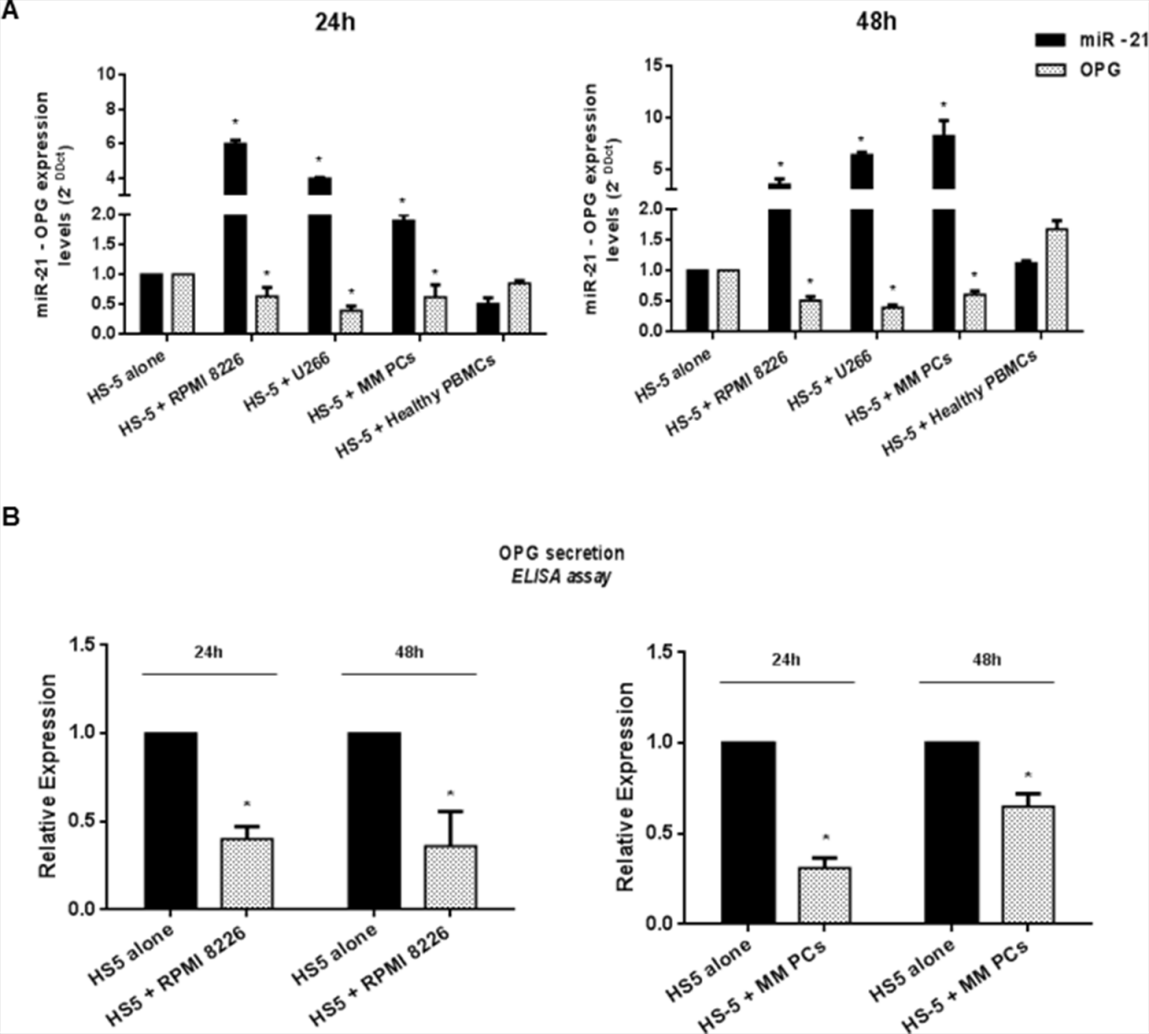
miR-21 upregulation in HS-5 correlates with OPG downregulation **A.** Quantitative RT-PCR analysis of miR-21 and OPG expression in HS-5 cultured alone (HS-5 alone) or adherent to either MM cell lines (HS-5 + RPMI 8226; HS-5 + U266) or primary MM cells (HS-5 + MM PCs) and exposed to healthy PBMCs (HS-5 + Healthy PBMCs). miR-21 expression increased by 6, 0-fold and 3, 46-fold in RPMI 8226 - HS-5 co-culture (*p* < 0.05), by 3, 9-fold and 6, 25-fold in U266 – HS-5 co-culture (*p* < 0.05) and by 2, 8-fold and by more than 8-fold (*p* < 0.05) in primary MM cells – HS-5 co-culture after 24 and 48 hours respectively. OPG expression significantly decreases in the presence of highest miR-21 expression levels (*p* < 0.05). Mean of Ct values were normalized to RNU44 housekeeping snoRNA or GAPDH and expressed as 2-DDCt value calculated using the comparative cross threshold method. Values represent mean ± SD of three independent experiments. **B.** ELISA analysis of OPG secretion in HS-5 cultured alone or co-cultured with RPMI 8226 or Primary MM cells. OPG concentration was reported as fold expression and each value, expressed in pmol/l, was normalized to HS-5 alone. Values represent the mean ± SD from three independent experiments. * indicates *p* < 0.05.

Our basic working hypothesis was that miRNA dysregulation in the BM *milieu* may account for OPG downregulation. At this aim, we first proceeded to identify putative miRNAs target sites on OPG 3′UTR by interrogating microRNA.org and TargetScan (version 6.2) data bases. Among predicted miRNAs, we focused on miR-221, miR-222 and miR-21, given their consolidated role as onco-miRNAs in MM [34, 35]. By qRT-PCR, we analyzed miR-221, miR-222 and miR-21 expression in the human HS-5 BM stromal cells cultured for 24 or 48 h with MM cells. No significant difference in miR-221 and -222 expression was detectable in HS-5 cultured with MM cells (Figure S2), while miR-21 expression significantly increased (*p* < 0.05) in HS-5 cultured with RPMI 8226 or U266 cells as compared to HS-5 cells cultured alone (Figure 1A). Upregulation of miR-21 was also found in HS-5 cultured with primary CD138^+^ cells from MM patients (Figure 1A) (*p* < 0.05) and in MM cells adherent to BMSCs (data not shown), as previous reported [34]. In parallel, we evaluated OPG production by qRT-PCR and ELISA assays in the same HS-5 culture conditions. As shown in Figure 1A and 1B, MM cells-induced miR-21 upregulation occurred together with a reduced OPG expression and secretion (*p* < 0.05). Importantly, HS-5 exposed to healthy PBMCs showed no miR-21 upregulation and OPG downmodulation (Figure 1A), further demonstrating that adherence to MM cells specifically promotes miR-21 overexpression in BMSCs. All together, these data suggest that the increase of miR-21 in BMSCs co-cultured with MM cells may play a role in downregulation of OPG.

### miR-21 is upregulated in MM patients-derived BMSCs

**Figure 2:**
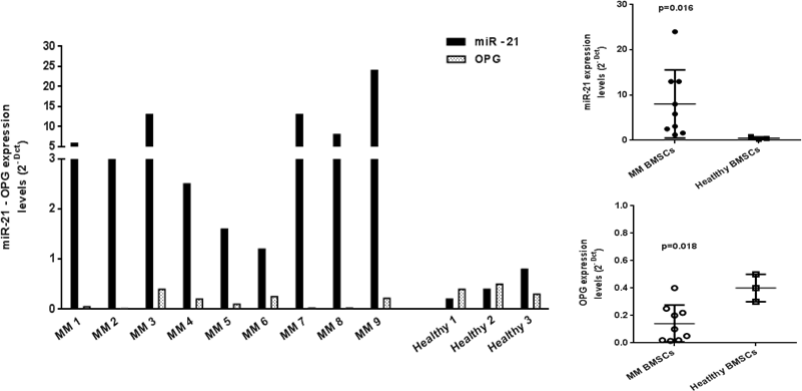
miR-21 is upregulated and OPG downregulated in MM patient-derived BMSCs Quantitative RT-PCR analysis of miR-21 and OPG from BMSCs of MM patients and from BMSCs of healthy donors after 3 weeks of culture period. Mean of Ct values were normalized to RNU44 housekeeping snoRNA or GAPDH and expressed as 2-DCt value calculated using the comparative cross threshold method. miR-21 expression levels of MM patients vs healthy donors: *p* = 0.016; OPG expression levels of MM patients vs healthy donors: *p* = 0.018.

To verify whether miR-21 might be a biomarker of MM-related BD, we analyzed by qRT-PCR miR-21 expression levels in BMSCs isolated from BM of MM patients and of healthy donors after 3 weeks of culture period. As reported in Figure 2, miR-21 was found dramatically overexpressed in almost all MM patients as compared to healthy individuals (*p* < 0.05). In parallel, we evaluated OPG expression in the same patient-derived BMSCs. We observed a marked OPG downregulation in MM BMSCs that showed highest miR-21 expression levels, thus indicating that our working hypothesis may be indeed true in the general disease context. Conversely, in healthy BMSCs miR-21 and OPG showed expression levels enough similar to each other (Figure 2).

### Enforced expression of miR-21 in HS-5 reduces OPG expression and secretion

**Figure 3:**
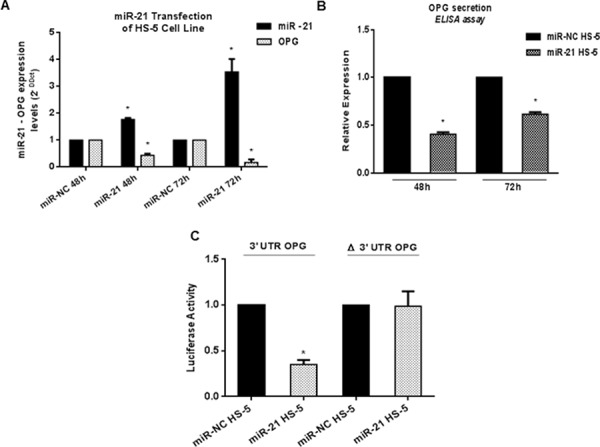
OPG expression is directly controlled by miR-21 **A.** Quantitative RT-PCR analysis of miR-21 and OPG expression in HS-5 transfected with synthetic miR-21 (miR-21 HS-5) or miRNA scrambled (miR-NC HS-5). Mean of Ct values were normalized to RNU44 housekeeping snoRNA or GAPDH and expressed as 2-DDCt value calculated using the comparative cross threshold method. Values represent mean ± SD of three independent experiments. **B.** ELISA analysis of OPG secretion calculated as fold expression. Each value expressed in pmol/l was normalized to control (miR-NC HS-5). Values represent mean ± SD of three independent experiments. **C.** Dual-luciferase assay of HS-5 co-transfected with firefly luciferase constructs containing the 3′UTR of OPG or its mutant lacking miR-21 target sequence (nts 601–675) and miR-21 or miRNA scrambled. The data are shown as relative luciferase activity of miR-21-transfected cells as compared with the control (miR-NC) of three independent transfections and were normalized to renilla luciferase activity. * indicates *p* < 0.05.

To assess if OPG production was really miR-21-dependent, we transfected HS-5 cells with miR-21 mimics or scrambled oligonucleotides (NC) and measured OPG expression by qRT-PCR and ELISA assays (Figure 3A and 3B). OPG mRNA expression decreased by 55% and 82% (*p* < 0.05) at 48 and 72 h, respectively (Figure 3A), and the secretion was dampened in miR-21 transfected HS-5 (miR-21 HS-5) as compared to miR-NC transfected cells (miR-NC HS-5) (Figure 3B) (*p* < 0.05).

To confirm that OPG mRNA was directly targeted by miR-21, we performed a luciferase assay by transfecting into HS-5 cells a reporter vector containing the 3′UTR sequence of OPG and a reporter vector containing the 3′UTR lacking the miR-21 binding site. As shown in Figure 3C, a marked reduction of luciferase activity (75%, *p* < 0.0001) was observed in cells transfected with the luciferase reporter vector together with synthetic miR-21 mimics, while no difference was seen in the presence of miR-NC. Conversely, mutation of the predicted miRNA binding site in the reporter vector abrogated this effect, indicating that miR-21 directly interacts with OPG 3′UTR.

These results demonstrate that the 3′UTR OPG mRNA is a direct target of miR-21 and suggest the relevance of a potentially druggable miR-21-OPG axis.

### Constitutive miR-21 inhibition significantly increases OPG production

**Figure 4:**
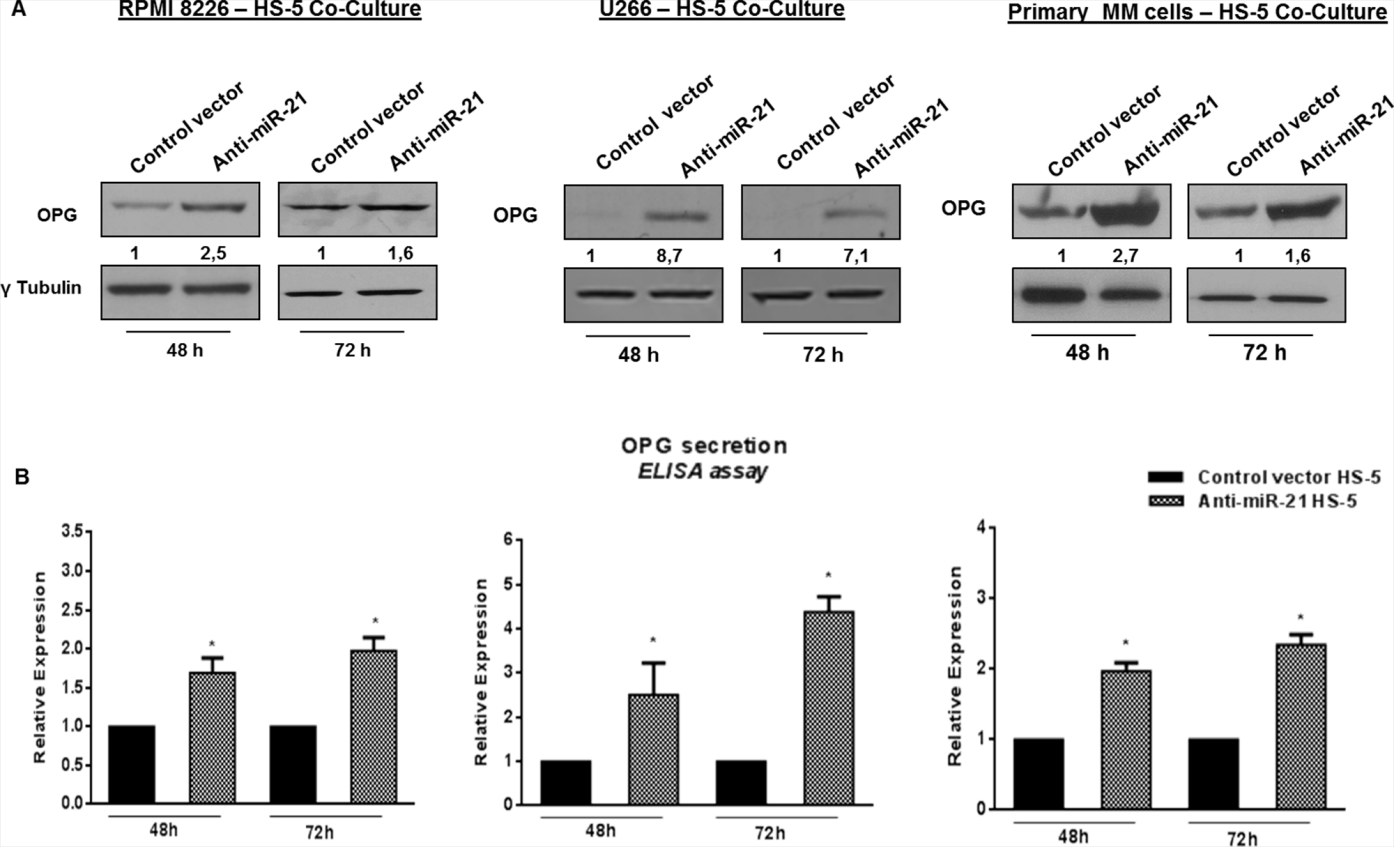
Constitutive miR-21 inhibition significantly increases OPG production **A, upper panels.** Immunoblot detection of OPG in anti-miR-21 and control vector HS-5 cultured for 48 and 72 hours with RPMI 8226, U266 and primary MM cells. γTubulin was used as loading control. **B, lower panels.** ELISA analysis of OPG secretion in RPMI 8226 – HS-5 co-culture, U266 – HS-5 co-culture, Primary MM cells – HS-5 co-culture. OPG concentration was reported as fold expression and each value, expressed in pmol/l, was normalized to control (control vector HS-5). Values represent the mean ± SD from three independent experiments. * indicates *p* < 0.05.

We next evaluated the effects of miR-21 inhibition on OPG production in HS-5 alone or adherent to MM cells. To this aim, HS-5 were transduced with a lentiviral vector carrying a miR-21 inhibitory sequence and a GFP reporter (anti-miR-21 HS-5) or with a lentiviral GFP-encoding vector (control vector HS-5) to obtain a stable miR-21 inhibition for all co-culture period. Then, HS-5 were cultured with either RPMI 8226 or U266 or primary CD138^+^ cells from MM patients. By qRT-PCR, we found that constitutive inhibition of miR-21 significantly increased OPG mRNA expression both in HS-5 alone (data not shown) and adherent to MM cell lines and primary MM cells (Figure S3, A, B and C) (*p* < 0.05). Next, we evaluated the OPG expression and secretion by western blotting and ELISA assays, respectively: indeed constitutive inhibition of miR-21 in HS-5 cells not only significantly increased OPG protein expression at both time points of co-cultures, but also restored OPG secretion in cell-culture medium (Figure 4A and 4B) (*p* < 0.05). Importantly, miR-21 antagonism considerably increased OPG secretion also in HS-5 cells cultured together with primary CD138^+^ cells from MM patients, consistently with results achieved with MM cell lines (Figure 4B, right panel) (*p* < 0.05). Taken together, these results demonstrate that constitutive miR-21 inhibition releases HS-5 from MM cell-induced inhibition of OPG expression and secretion in the BM *milieu*.

### Constitutive inhibition of miR-21 decreases RANKL production

**Figure 5:**
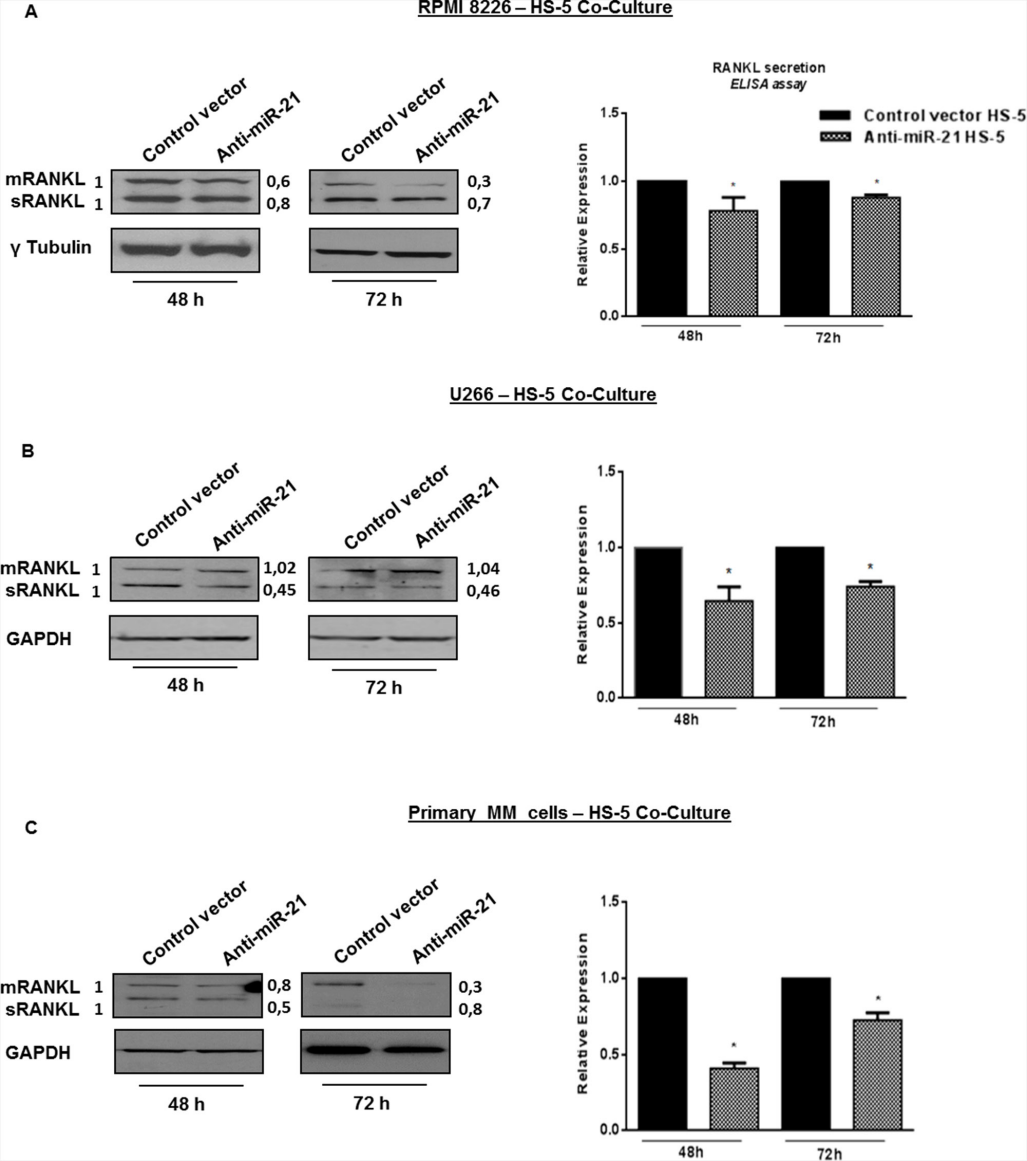
Constitutive miR-21 inhibition reduces RANKL production **A, B and C left panels.** Immunoblot detection of mRANKL (transmembrane RANKL) and sRANKL (soluble RANKL) in anti-miR-21 and control vector HS-5 cultured for 48 and 72 hours with RPMI 8226 (A), U266 (B) and primary MM cells (C) γTubulin was used as loading control for RPMI 8226, GAPDH for U266 and primary MM cells – HS-5 co-cultures. A, B and C right panels. ELISA analysis of RANKL secretion in RPMI 8226 (A), U266 (B) and primary MM cells (C) – HS-5 co-cultures. RANKL concentration was calculated as fold expression and each value, expressed in pmol/l, was normalized to control. Values represent mean ± SD of three independent experiments. * indicates *p* < 0.05.

Although the increase of OPG within BM *milieu* may restore RANKL/OPG balance by itself, a corresponding reduction in RANKL secretion would further limit the osteolytic property of BMSCs stimulated by MM cell contact. On these premises, we studied if miR-21 inhibition might also interfere with RANKL production. Indeed antagonism of miR-21 reduced RANKL mRNA (data not shown), both sRANKL (soluble RANKL) and mRANKL (transmembrane RANKL) protein expression levels in HS-5 adherent to MM cell lines (Figure 5A and 5B, left panels) as well as sRANKL secretion in cell-culture medium (Figure 5A and 5B, right panels) (*p* < 0.05). A rescue in RANKL production was observed at 72 h of co-culture period probably because sRANKL mostly accumulates in cell-culture medium as compared to 48 h. These findings are of special interest because it has been demonstrated that RPMI 8226 cells impair RANKL/OPG ratio when cultured with BMSCs or OBLs [45].

Importantly, we confirmed these observations also when HS-5 were adherent to primary CD138^+^ cells from MM patients. Again, antagonism of miR-21 significantly reduced both isoforms of RANKL protein as well as sRANKL secretion (*p* < 0.05) (Figure 5C). Altogether, these data indicate that inhibition of miR-21 restores the physiologic RANKL/OPG balance.

### Constitutive inhibition of miR-21 restores RANKL/OPG ratio in co-cultures of MM patient-derived BMSCs

**Figure 6:**
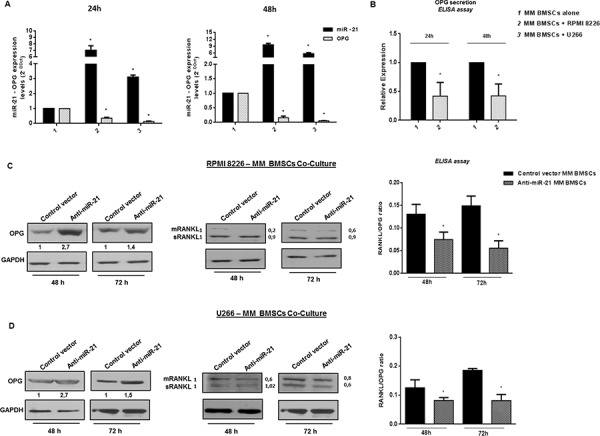
miR-21 is upregulated in primary patient BMSCs adherent to MM cells and its constitutive inhibition restores RANKL/OPG ratio **A.** Quantitative RT-PCR analysis of miR-21 and OPG expression in primary patient BMSCs alone (MM BMSCs alone) and adherent to either MM cell lines (MM BMSCs + RPMI 8226; MM BMSCs + U266). miR-21 expression increased by 7-fold and 9, 5-fold in RPMI 8226 - MM BMSCs co-culture (*p* < 0.05), 3, 1-fold and 5, 6-fold in U266 - MM BMSCs co-culture (*p* < 0.05) after 24 and 48 hours respectively. OPG expression significantly decreases in the presence of highest miR-21 expression levels (*p* < 0.05). Mean of Ct values were normalized to RNU44 housekeeping snoRNA or GAPDH and expressed as 2-DDCt value calculated using the comparative cross threshold method. Values represent mean ± SD of three independent experiments. **B.** ELISA analysis of OPG secretion in primary patient BMSCs cultured alone or with RPMI 8226. OPG concentration was reported as fold expression and each value, expressed in pmol/l, was normalized to MM BMSCs alone. Values represent the mean ± SD from three independent experiments. **C and D, left panels.** Immunoblot detection of OPG in anti-miR-21 and control vector MM BMSCs cultured for 48 and 72 hours with RPMI 8226 (C) and U266 (D) GAPDH was used as loading control. **C and D central panels.** Immunoblot detection of mRANKL (transmembrane RANKL) and sRANKL (soluble RANKL) in anti-miR-21 and control vector MM BMSCs cultured for 48 h and 72 h with RPMI 8226 (C) and U266 (D) GAPDH was used as loading control. C and D, right panels. ELISA analysis of OPG and RANKL concentration expressed in RANKL/OPG ratio in RPMI 8226 (C) and U266 (D) – MM BMSCs co-cultures. Each value of RANKL (pmol/l) was divided to each value of OPG (pmol/l). Values represent mean ± SD of three independent experiments. *indicates *p* < 0.05.

We next cultured primary MM patient-derived BMSCs with MM cells. As shown in Figure 6A, increased levels of miR-21 were found in MM BMSCs after 24 or 48 hours of co-culture (*p* < 0.05). Consistently with results achieved from HS-5 cell line, also MM BMSCs adherent to MM cells produce low level of OPG, as reported in Figure 6A and 6B. MM BMSCs were then transduced with miR-21 inhibitors and cultured with RPMI 8226 or U266 cells: effects on OPG production were evaluated by qRT-PCR, western blotting and ELISA assays. Notably, increased OPG mRNA (Figure S5, A and B) (*p* < 0.05) and protein levels, along with a marked reduction of both mRANKL and sRANKL, were observed in cells transduced with miR-21 inhibitors and adherent to RPMI 8226 and to U266 (Figure 6C and 6D), further emphasizing the role of miR-21 inhibition in restoring RANKL/OPG balance.

Finally, by ELISA assay we analyzed OPG and RANKL secretion in cultures of MM cells adherent to either anti-miR-21 or control vector MM BMSCs. Constitutive inhibition of miR-21 in MM BMSCs significantly decreased RANKL/OPG ratio into culture medium (Figure 6C and 6D, right panels), due to both reduced RANKL and increased OPG secretion levels. Since RANKL/OPG imbalance is the dominant and final mediator of bone resorption, this finding on primary MM patient-derived BMSCs further reinforces the value of miR-21 as one of key players in MM-related BD.

### Inhibition of miR-21 suppresses RANKL through PIAS3 upregulation

**Figure 7:**
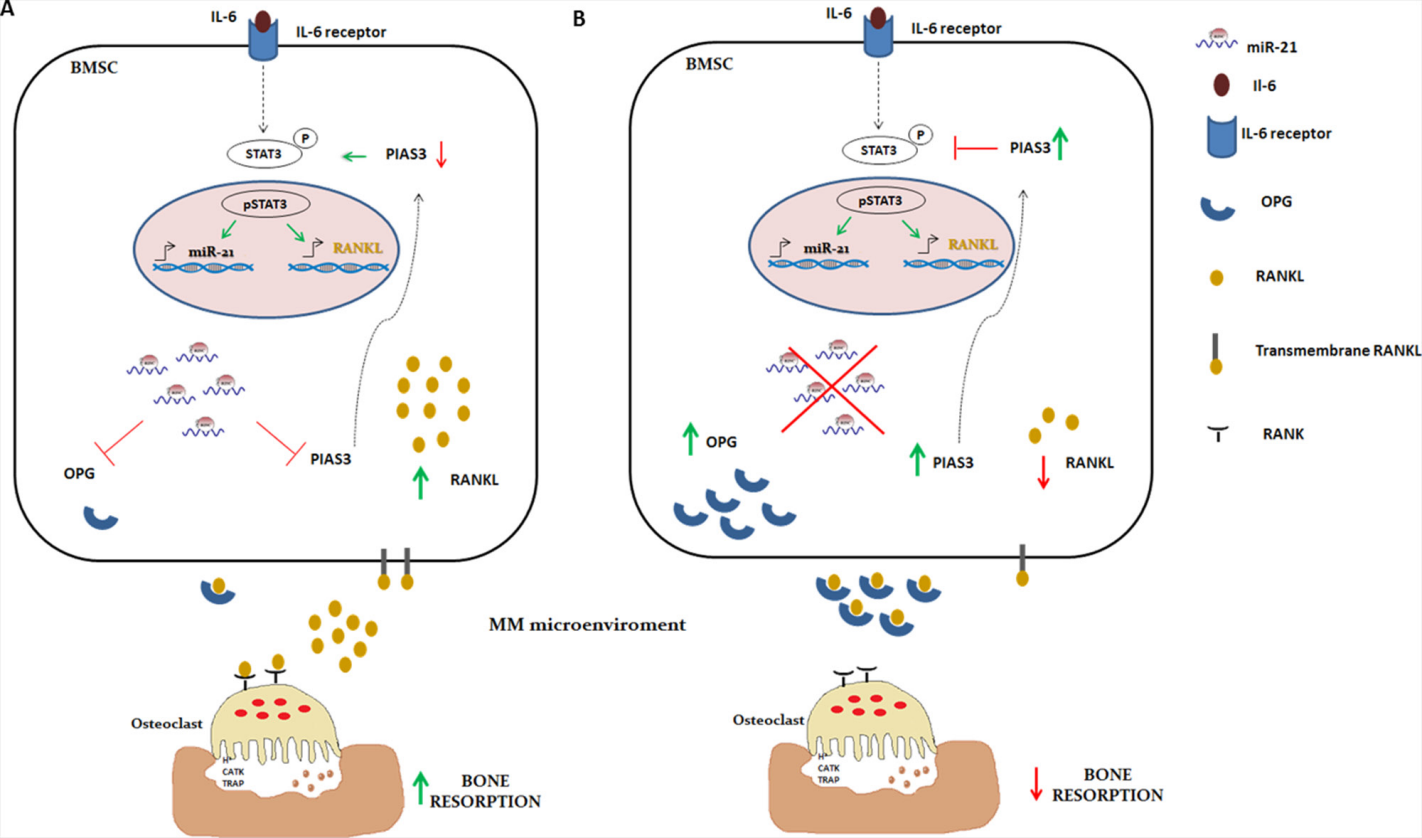
Descriptive cartoon of IL-6, miR-21 and STAT3 axis **A.** IL-6 signaling leads to RANKL and miR-21 expression in BMSCs through STAT3 activation. miR-21 overexpression inhibits OPG production and, in a positive feedback loop, promotes RANKL gene activation by reducing PIAS3, specific inhibitor of active STAT3 (pSTAT3). The RANKL/OPG imbalance in MM microenviroment results in a severe perturbation of bone homeostasis. **B.** Inhibition of miR-21 restores PIAS3 and OPG expression: PIAS3 interferes with RANKL gene expression by inhibiting STAT3 activation, while in MM microenviroment OPG antagonizes RANKL binding on this receptor RANK, thus restoring the physiologic OCLs activity.

**Figure 8:**
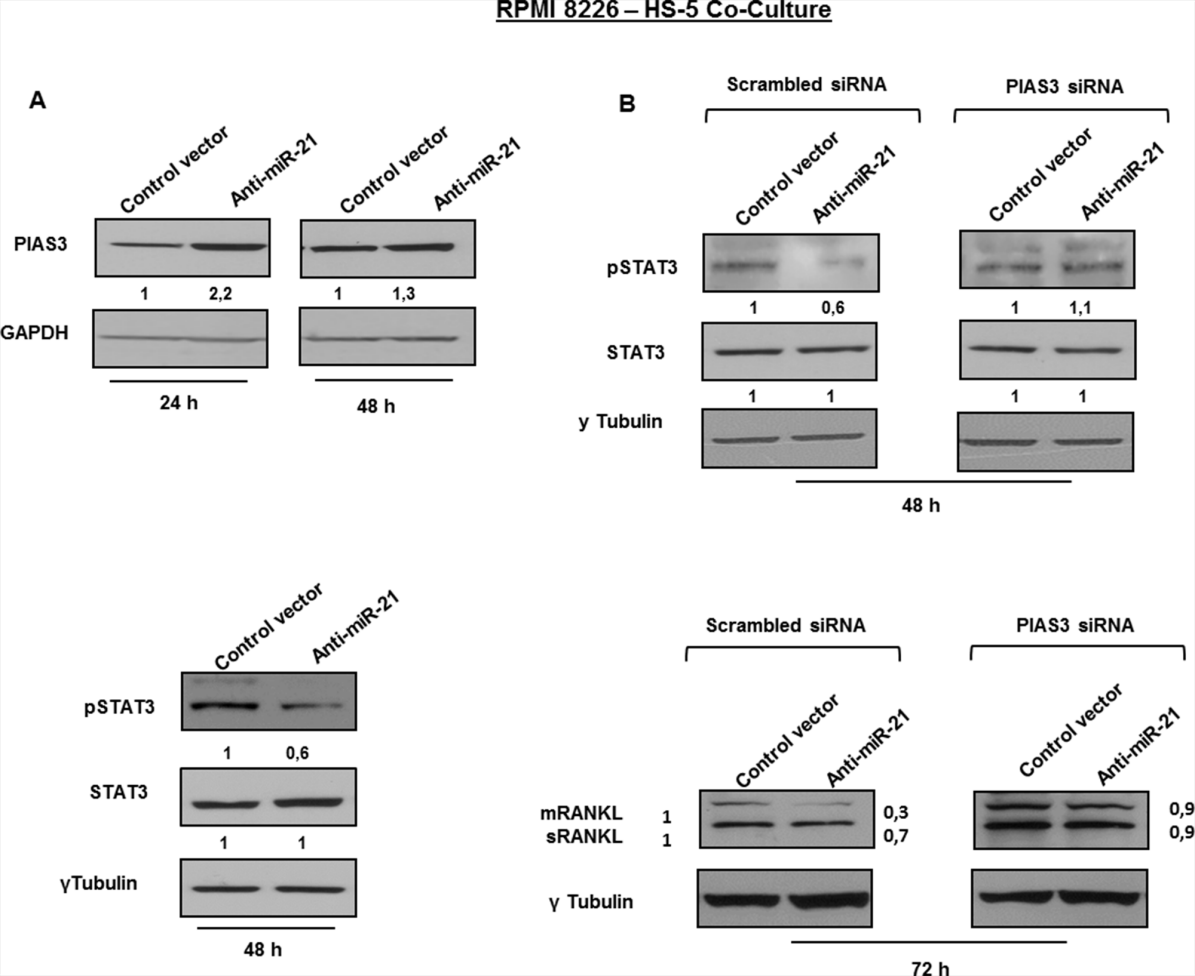
Inhibition of miR-21 suppresses RANKL through PIAS3 upregulation **A.** Immunoblot detection of PIAS3 (upper panel) and pSTAT3 (lower panel) in anti-miR-21 and control vector HS-5 cultured with RPMI 8226. GAPDH and γTubulin were used as loading control. **B.** Immunoblot detection of pSTAT3 (upper panel) and mRANKL (transmembrane RANKL) and sRANKL (soluble RANKL) (lower panel) in anti-miR-21 and control vector HS-5 transfected with specific PIAS3 or scrambled stealth siRNAs and cultured with RPMI 8226. γTubulin was used as loading control.

Protein inhibitor of activated STAT3 (PIAS3), a validated target of miR-21 [46, 47], negatively regulates phosphorylation and activation of signal transducer and activator of transcription 3 (STAT3). Importantly, IL-6 induces the expression of both miR-21 and RANKL by signaling through STAT3 [41, 48], as described in the cartoon reported in Figure 7A. These considerations led us to evaluate whether lower expression of RANKL in BMSCs stably expressing miR-21 inhibitors could be ascribed to PIAS3-dependent inhibition of STAT3 signaling (Figure 7B). At this aim, we firstly confirmed that ectopic expression of miR-21 mimics effectively downregulated PIAS3 in HS-5 cells (Figure S7, A and B) and next, we evaluated the effects of miR-21 inhibition. Indeed, PIAS3 expression was increased at both mRNA (Figure S6 A, B and C) and protein levels (Figure 8A, upper panel) in cells transduced with miR-21 inhibitors in all co-culture systems (*p* < 0.05). Furthermore, significantly reduced STAT3 phosphorylation occurs in the same experimental settings (Figure 8A, lower panel). To prove that RANKL downregulation was indeed PIAS3-dependent, we knocked-down PIAS3 expression by transfecting anti-miR-21- or control vector -transduced HS-5 cells with specific PIAS3 siRNAs. Effective silencing of PIAS3 was confirmed as reported in Figure S7, B, C and D. Next we performed co-culture experiments with MM cells. As shown in Figure 8B (lower panel), no difference in RANKL expression was observed in either anti-miR-21- nor control vector -transduced cells when PIAS3 was suppressed by specific siRNA. Accordingly, silencing of PIAS3 restored pSTAT3 in anti-miR-21 transduced-HS-5 cells (Figure 8B, upper panel).

All together, these findings clearly support the notion that downregulation of RANKL in the presence of miR-21 antagonism occurs in a PIAS3- and pSTAT3-dependent manner.

### Co-culture medium from HS-5 stably expressing miR-21 inhibitors suppresses OCL activity *in vitro*

**Figure 9:**
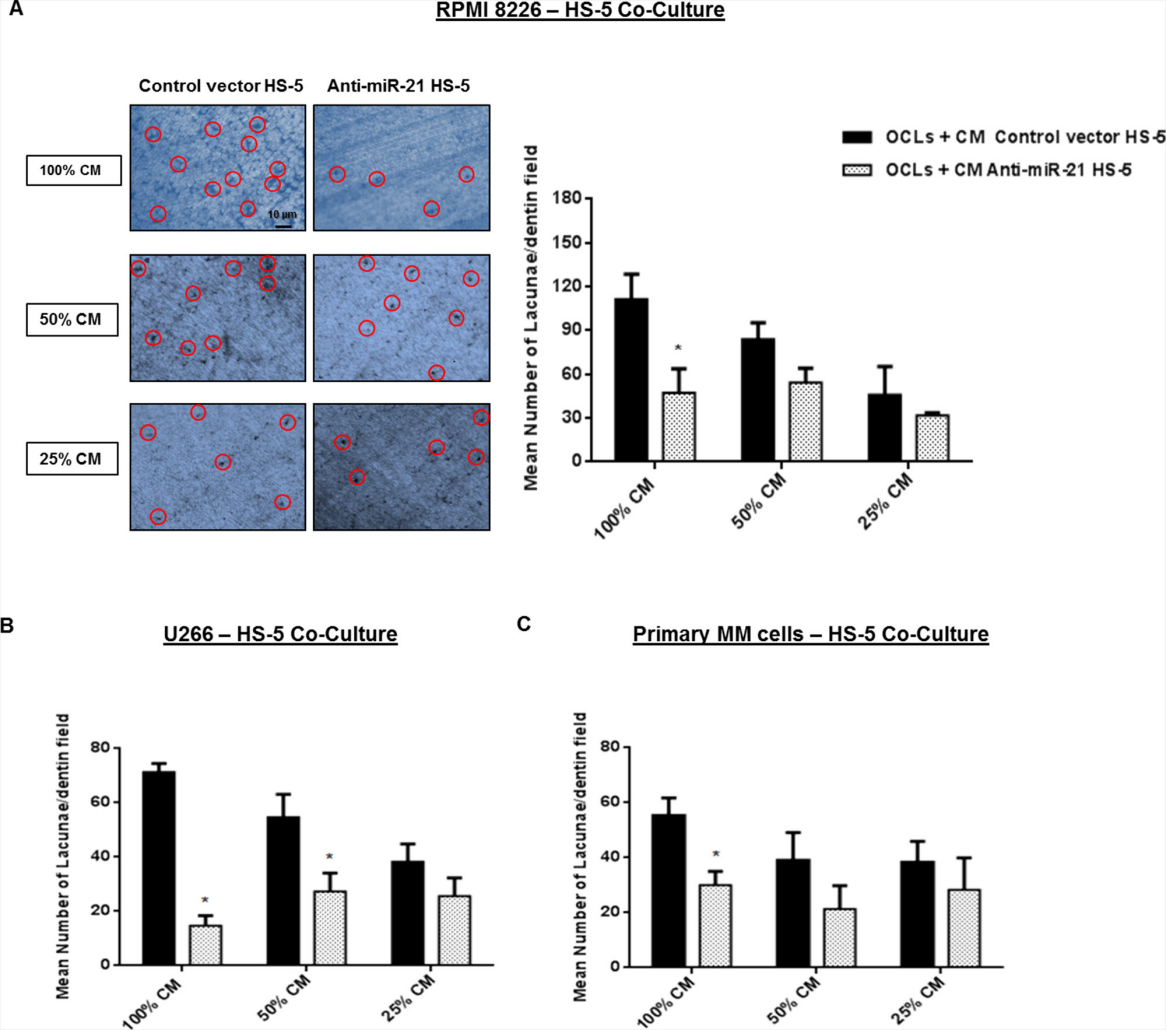
Exposure to co-culture medium from HS-5 stably expressing miR-21 inhibitors suppresses the OCLs activity **A, left panel.** Analysis of lacunae generation on dentin surfaces of OCLs cultured for 4–5 days with serial dilutions of culture medium (100% CM, 50% CM and 25% CM) form RPMI 8226 – control vector or anti-miR-21 - HS-5 co-culture (OCLs + CM control vector HS-5; OCLs + CM anti-miR-21 HS-5). Red circles showed on representative dentin surfaces indicate some single bone resorption lacunae identified by blue toluidine staining. A, right panel, **B and C.** Analysis of lacunae generation by NIH *imageJ* software that considered each pit as a dark area on dentin surface. Results shown are the mean from lacunae number in three dentin fields out of three independent experiments. *indicates *p* < 0.05.

We next investigated whether the increased OPG secretion resulted in impaired OCL-bone resorption activity. To this aim, we seeded OCLs on dentin slices upon exposure to RANKL and M-CSF for 14 days. Then, we stimulated final OCLs maturation by adding medium obtained from transduced-HS-5 cultured with either MM cell lines or primary CD138^+^ MM cells. In Figure 9A (left panel) are shown representative dentin surfaces of OCLs exposed to medium from RPMI 8226 - HS-5 co-cultures. When OCLs were exposed to the highest amount of medium collected from anti-miR-21 HS-5 co-cultures, their ability to degrade dentin surfaces was markedly reduced (*p* < 0.0001) as compared to OCLs exposed to medium collected from control vector HS-5 co-cultures (Figure 9A, 9B and 9C). Serial dilutions of culture medium progressively abrogated this effect.

These results indicate that the balancing activity induced by miR-21 inhibition on OPG and RANKL secretion in co-cultures medium results in bone resorption antagonism.

## DISCUSSION

In the last years, the clinical outcome of MM patients have significantly improved by active anti-MM drugs including bortezomib or lenalidomide [49] and novel translational platforms have recently opened new areas of investigation [50–58]. Nevertheless, MM is still a lethal disease with high morbidity for patients, including its related skeletal destruction. BD in fact is a highly symptomatic condition and has a relevant impact on disease outcome. Many factors are involved in the physiological balance between bone apposition and resorption. MM-related BD is produced by interaction of MM cells with BMSCs, that are induced to secrete high levels of RANKL [2], a critical osteoclastogenic factor, and low levels of OPG [4], the main bone protective factor. This condition results in RANKL/OPG imbalance and in bone destruction. Based on the emerging evidence of miRNAs as key players in bone remodelling [27, 59], we investigated if interference with the miRNA-network within BMSCs, might counteract MM-induced OPG inhibition, finally restoring the physiologic RANKL/OPG balance. In this light, we first identified miRNAs targeting the 3′UTR of OPG, then we evaluated their modulation in response to MM cell contact. Among putative OPG-targeting miRNAs evaluated, miR-21 only was highly upregulated in BMSCs, the most relevant microenviroment-related OPG producing cells, in adherence to MM cells. Furthermore, OPG displayed lower levels when miR-21 was upregulated while enforced miR-21 expression downregulated OPG in HS-5 BMSCs. The miR-21-specific OPG targeting was indeed validated by luciferase assay. To our knowledge, this is the first report demonstrating the specific 3′UTR targeting of OPG by miR-21. Importantly, miR-21 is abnormally expressed in MM patient-derived BMSCs together with very low OPG expression. These findings strongly indicate miR-21 as a favorite trigger element of the MM microenviroment-induced OPG downmodulation.

Hence, we speculated that miR-21 inhibition could abrogate OPG downregulation induced by MM cells contact. To verify our hypothesis, we cultured MM cells with HS-5 BMSCs stably expressing miR-21 inhibitory sequences. As expected, miR-21 antagonism restored OPG production thus overcoming the inhibitory effect produced by direct interaction between MM cells and BMSCs. As further proof of the bone-protective role of miR-21 inhibition, we demonstrated that miR-21 antagonism was also effective in preventing MM-induced secretion of RANKL by HS-5 BMSCs. We think that these findings are highly relevant for understanding MM-related BD since selective inhibition of miR-21 aberrantly expressed in BMSCs led to recovery of the physiological secretion of both RANKL and OPG.

It has been demonstrated that RANKL production is under a regulatory feedback loop involving miR-21, IL-6 and STAT3 pathways [41, 60], while miR-21 expression is induced by IL-6 and requires STAT3 pathway activation [41, 61]. In turn miR-21 enhances the STAT3-dependent signaling by inhibiting PIAS3, a validated miR-21 target in human malignancies [46, 47]. In BMSCs and OBL cells, RANKL gene activation is under the direct control of pSTAT3 through IL-6 signaling activation [62]. Since PIAS3 is a specific inhibitor of STAT3 phosphorylation [63], it interferes with STAT3-mediated RANKL expression [48]. Recently, it has been also demonstrated by others that RANKL-induced osteoclastogenesis is repressed by silencing miR-9718, a specific inhibitor of PIAS3 [59].

On this evidence, we speculated that antagonizing miR-21 in our experimental system may result in a reduced STAT3 signaling mediated by PIAS3 upregulation. Accordingly with our working hypothesis, high levels of PIAS3 and low levels of phosphorylated STAT3 were indeed found in HS-5 BMSCs stably expressing miR-21 inhibitors. Direct proof of the PIAS3 inhibitory effect on RANKL expression was provided by the finding that siRNA-mediated PIAS3 silencing resulted in the loss of RANKL downmodulation induced by miR-21 inhibition which occurred together with reactivation of STAT3 signaling. Finally, we demonstrated that miR-21 inhibition impairs OCLs cell function: the resorptive activity of mature OCLs exposed to anti-miR-21 HS-5 co-cultures medium was in fact dramatically reduced. In a previous report [27], we provided evidence that constitutive expression of miR-29b negatively regulates OCL differentiation and function by reducing intracellular levels of specific resorbing enzymes. In the present work, we provide additional information on miRNA role in OCL regulation by demonstrating that miR-21 overexpression induced by MM-BMSCs interaction antagonizes the physiologic RANKL/OPG balance and that OCLs activity is tightly dependent by BMSCs miRNA-network perturbation. All together, our findings strongly highlight the anti-MM value of miR-21 inhibitory approaches and support the notion that combination of miR-21 antagonism with conventional drugs might improve the clinical outcome of MM patients. In this view, further investigations of miR-21 inhibition by a suitable *in vivo* model of MM BD are need to open a way for clinical investigation of miR-21 antagonists-based therapies in the management of MM-related BD.

## MATERIALS AND METHODS

A detailed description of reagents, cell culture and experimental procedures is provided in Supplementary Methods.

### Cell lines, primary MM cells and co-culture conditions

RPMI 8226 and U266 MM cell lines and the healthy bone marrow stromal cell line HS-5 were purchased from American Type Culture Collection (ATCC, Rockville, MD, USA) and cultured as described in Supplementary Methods.

After informed consent approved by our University Hospital Ethical Committee, primary MM cells were isolated from MM patient by Ficoll-hypaque separation method (Lonza Group, Basel, Switzerland), followed by antibody mediated positive selection using anti-CD138 microbeads kit (Milteny Biotech, Gladbach, Germany). MM BMSCs were isolated by negative selection of BM aspirates from MM patients while healthy BMSCs were isolated from BM cells derived from skeletal fragments of healthy subjects and long-term cultured in RPMI1640 supplemented by 20% FBS and 1% penicillin/streptomycin (Gibco, Life Technologies, Carlsbad, CA) [51]. Healthy peripheral blood mononuclear cells (PBMCs) were isolated from peripheral blood of healthy subjects by Ficoll-hypaque separation method (Lonza Group, Basel, Switzerland).

Co-culture experiments were performed in 6 well plate at a density of 2,5 × 10 ^5^ cells/ ml in 1:1 HS-5 (or MM BMSCs)/MM cells ratio. Healthy PBMCs co-cultures experiments were performed in a 6 well plate by using Transwell insert of 0, 4 μm pore size (Corning, New York, USA) in 1:10 HS-5/PBMCs ratio. We used U266 as IL-6 secreting cell line [64] and RPMI 8226 given their ability to upregulate RANKL and downregulate OPG expression in OCL precursors or stromal cells co-cultures [45]. After 24, 48 or 72 h of co-culture, stromal cells (either HS-5 or MM BMSCs) were harvested following depletion of MM CD138^+^ cells using anti-human CD138 microbeads kit (Milteny Biotech, Gladbach, Germany). As shown in Figure S1, only samples with >95% purity were selected for RNA and protein isolation.

### Construction of lentiviral vectors and stromal cells infection

To attain cells stably expressing miR-21 inhibitory sequences, HS-5 or MM BMSCs were transduced with the miR-Zip-21 anti-miR-21 lentiviruses (System Biosciences, Mountain View, CA) produced in 293Ta cells [65]. Two days after transduction, selection with 0, 5 μg/ml puromycin was performed and 95% of transduction efficiency, evaluated by flow cytometry analysis (Figure S1), was generally obtained in HS-5 cells. After 4–5 days of puromycin selection, HS-5 cells were ready for co-culture experiments.

Transduction efficiency of MM BMSCs was monitored by investigating the percentage of green fluorescent protein (GFP) positive cells using a fluorescence microscope (Figure S4).

### 3′UTR luciferase reporter assays

The 3′UTR of OPG or its mutant lacking miR-21 target sequence (nts 601–675) were cloned in pEZX-MT01 vector and purchased from Genecopoeia (Rockville, MD, USA). HS-5 cells were electroporated, as described below, using 5 μg of the firefly luciferase reporter construct and 100nM of miR-21 mimics or scrambled miR-negative control (NC). Renilla luciferase activities were measured to normalize expression of firefly luciferase. Firefly and Renilla luciferase activities were measured 48 h after transfection using the dual-luciferase assay kit (Promega, Madison, WI) following the manufacturer's instructions.

### *In vitro* transfection of HS-5 cells

Synthetic miR-21 mimics (#MC10206) and scrambled miR-negative control (miR-NC) were purchased from Ambion (Applied Biosystems), silencer selected stealth siRNAs for PIAS3 (#HSS115924, #HSS115925, #HSS173612) and scrambled stealth siRNAs control were purchased from Invitrogen (Life technologies, Carlsbad, CA). Synthetic miR-21 mimics or miR-NC were transfected to final concentration of 100 nM, while 90 nM was the final concentration for selected silencer siRNAs. A total 2,5 × 10^5^ HS-5 cells were transfected using Neon Transfection system (Invitrogen) applying the same conditions for each transfection experiment (2 pulses at × 1300V, 20 milliseconds). Efficiency of cells transfection was evaluated by quantitative real time PCR (qRT-PCR) (Figure 3A).

### RNA isolation and quantitative Real Time (qRT)- PCR

See Supplementary Methods for detailed experimental procedure and data analysis.

### Western blotting

Western blotting was performed as described [66]. Briefly, HS-5 or MM BMSCs were lysed in NP40 CellLysis Buffer (Novex^®^) containing a cocktail of protease inhibitors (Sigma Aldrich, Steinheim, Germany). Whole cells lysates (30 μg per lane) were separated using 4–12% Novex Bis-Tris SDS-acrylamide gels (Gibco, Life Technologies, Carlsbad, CA), electro-transferred on Nitrocellulose membranes by Trans-Blot^®^ Turbo™ Transfer Starter System for 7 min and immunoblotted with the primary antibodies. The following antibodies were used: OPG (#ab14049) from Abcam (Cambridge, UK), RANKL (#4816), PIAS3 (#4164), STAT3 (#9145) and pSTAT3 (#9131) from Cell Signaling Technology (Danvers, MA), GAPDH (sc-25778) and γ-Tubulin (sc-17787) from Santa Cruz Biotechnology (Santa Cruz, CA). Two types of loading control were used to avoid bands overlapping. Goat anti-mouse and goat anti-rabbit antibodies HRP-conjugated (Santa Cruz Biotechnology) were used as secondary antibodies.

Immunoreactive bands were detected by using the enhanced chemiluminescence (ECL) method (Santa Cruz Biotechnology, Santa Cruz, CA). The protein bands were scanned and quantified by using NIH *ImageJ analysis program*.

### ELISA assay

OPG and sRANKL secretion in cell culture medium of all co-culture systems were measured by ELISA using kits from BioVendor (#RD194003200, Modrice, Czech Republic) and Biomedica GmbH (#BI20452, Vienna, Austria), respectively.

### Collection and preservation of co-culture media

Culture Medium (CM) of lentiviral transduced-HS-5 cultured for 48 and 72 h with either MM cell lines or primary CD138^+^ from MM patients was collected, centrifugated at 1200 rpm for 5 min, filtered through a 0.22 μm syringe filter and conserved at −80°C until use. Once evaluated the concentrations of OPG and sRANKL by ELISA assay, CM of either RPMI 8226, U266 or primary MM cells – HS-5 co-cultures, was diluted at 0%, 50% or 75% (for 100%, 50% and 25% CM respectively) (Figure 9A, 9B and 9C) with αMEM supplemented by 10%FBS and 1% penicillin/streptomycin (Gibco, Life Technologies, Carlsbad, CA) and used as culture medium of OCLs for 4–5 days as described in Methods.

### Bone resorption detection assay

After 14 days of culture in OCL differentiation medium, OCLs were removed from 6 well plates according to previously established protocols [27] and seeded on organic dentine discs (#AE8050, Pantec, Turin, Italy) at a density of 1 × 10^4^ /ml. For additional 4–5 days, OCLs on dentin slices were cultured with serial dilutions of HS-5 co-cultures medium. Following blue toluidine staining, lacunae generation was measured by NIH *ImageJ* imaging software, considering each resorptive pit as a dark area on dentin surface. At least three fields of each dentin disc were evaluated in three independent experiments (at 20x magnification).

### Statistical analysis

Each experiment was performed at least 3 times and all values are reported as means ± SD. Comparisons between groups were made with student's *t*-test by using Graphpad Prism version 6.0 statistical package.

Differences were considered statistically significant if *p*-value was lower than 0.05.

## SUPPLEMENTARY DATA FIGURES


