# Supplementary material for: Inhibition of miR-21 restores RANKL/OPG ratio in multiple myeloma-derived bone marrow stromal cells and impairs the resorbing activity of mature osteoclasts
Source: Oncotarget. 2015 Jun 24;6(29):27343–58. doi: 10.18632/oncotarget.4398 (PMC4694994; doi:10.18632/oncotarget.4398)
Supplement: Supplementary file 1 [file oncotarget-06-27343-s001.pdf]

SUPPLEMENTARY DATA

Cell cultures

RPMI 8226 and U266 were cultured in RPMI1640 (Gibco, Life Technologies, Carlsbad, CA) supplemented with 10% and 20% FBS respectively and 1% penicillin/streptomycin (Gibco, Life Technologies, Carlsbad, CA), while HS-5 were cultured in DMEM (Gibco, Life Technologies, Carlsbad, CA) supplemented with 10% FBS and 1% penicillin/streptomycin (Gibco, Life Technologies, Carlsbad, CA).

Flow-cytometric analysis

Flow-cytometric analysis was used to assess purity of immune selected cells ($CD138^+$ cells), of HS-5 BMSCs harvested following depletion of MM $CD138^+$ cells and to assess transduction efficiency of HS-5 (Figure S1).

Phycoerythrin-conjugated CD138 monoclonal antibody (Milteny Biotech, Gladbach, Germany) was used to assess purity of immune selected cells.

Finally, the transduction efficiency was evaluated as percentage of cells expressing GFP through FACS Calibur examination.

RNA isolation and quantitative Real Time (qRT)-PCR

Total RNA was isolated using TRIzol® reagent (Gibco, Life Technologies, Carlsbad, CA) following the manufacturer's instructions. The RNA's quantity and quality was evaluated through NanoDrop® (ND-1000 Spectrophotometer) choosing samples with 260\280 absorbance ratio > 1, 8. To evaluate miRNA-21 expression level, 10 ng of total RNA were reverse transcribed to cDNA using "TaqMan micro-RNA reverse transcription kit" (Applied Biosystems, Carlsbad, CA). To evaluate the OPG, RANKL and PIAS3 gene expression levels, 2 μg of total RNA were reverse transcribed to cDNA using "High Capacity cDNA Reverse Transcription Kit" (Applied Biosystems, Carlsbad, CA).

The single-tube TaqMan miRNA assays (Applied Biosystems, Carlsbad, CA) was used to detect and quantify mature miR-21 (assay ID: 000397), miR-221 (assay ID: 000524), miR-222 (assay ID: 000525) and the other genes according to the manufacturer's instructions, using Viia 7 Dx multicolor detection system (Applied Biosystems, Carlsbad, CA). The obtained Threshold Cycle (CT) values were normalized on RNU44 (assay ID: 001094) for miR-21, -221, -222 and on GAPDH (Hs03929097_g1) for OPG (Hs00900538_m1), RANKL (Hs00243522_m1) and PIAS3 (Hs00180666_m1).

Comparative real-time polymerase chain-reaction (RT-PCR) was performed in triplicate, including no-template controls. Relative expression was calculated using the comparative cross threshold (Ct) method.

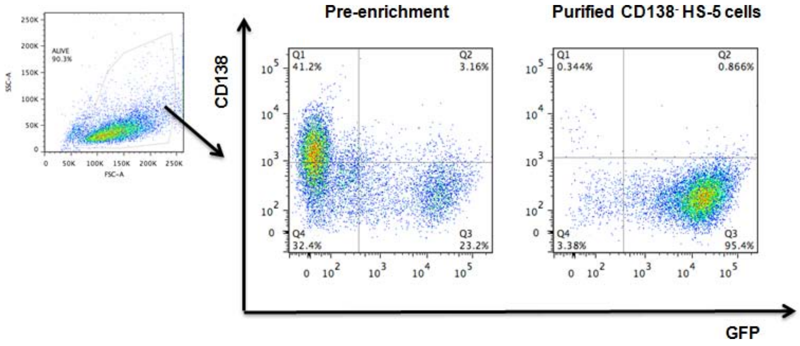

Supplementary Figure S1: Flow cytometry analysis of HS-5 purity after MM CD138+ cells depletion. Representative image showing purity of HS-5 after negative selection by magnetic column separation from HS-5 co-cultures. Cells obtained from co-cultures were incubated with anti-human CD138 microbeads and passed through a magnetic column. A small aliquot was taken before (left panel) and after (right panel) enrichment to evaluate purity. Percent of alive cells from initial gating is also shown (upper panel, left).

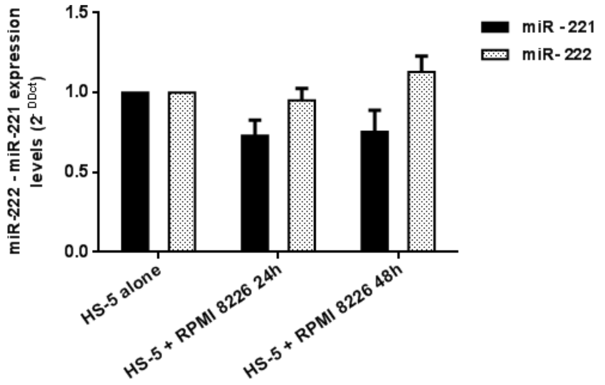

Supplementary Figure S2: RT-PCR analysis of miR-221 and miR-222 in HS-5 co-cultured with RPMI 8226 MM cell line. Quantitative RT-PCR analysis of miR-221 and miR-222 in HS-5 cultured alone or with RPMI 8226 MM cells. No statistical difference was observed in miR-221 and -222 expression levels between HS-5 alone and adherent to RPMI 8226 MM cells for 24 and 48 hours. Mean of Ct values were normalized to RNU44 housekeeping snoRNA or GAPDH and expressed as 2-DDCt value calculated using the comparative cross threshold method. Values represent mean ± SD of three independent experiments.

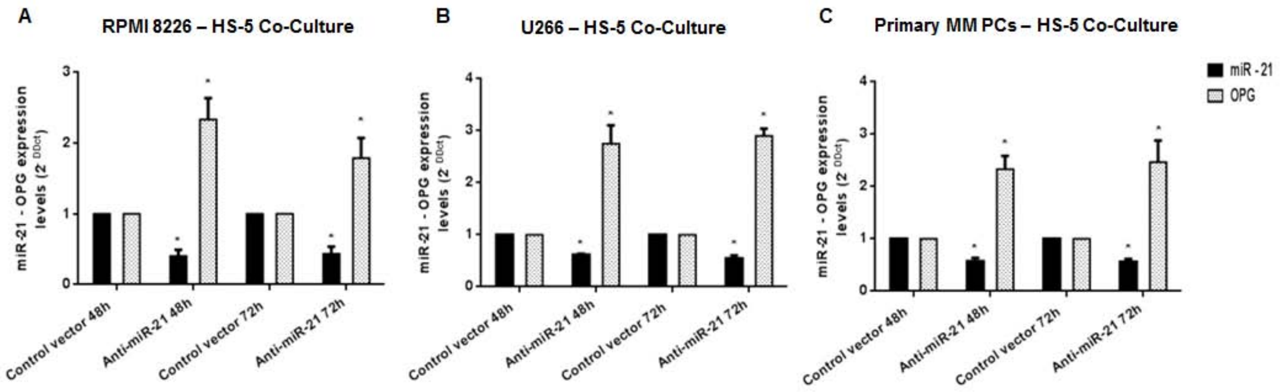

Supplementary Figure S3: RT-PCR analysis of OPG mRNA in HS-5 stably expressing miR-21 inhibitory sequences. Quantitative RT-PCR analysis of miR-21 and OPG in HS-5 transduced with a lentiviral vector carrying miR-21 inhibitory sequences (anti-miR-21 HS-5) or GFP reporter gene (control vector HS-5) and adherent to either RPMI 8226 or U266 or primary MM cells. OPG mRNA levels increased by 2, 4-fold and 1, 8-fold in **A.** RPMI 8226 - HS-5 co-culture (p <0.05), 2, 75-fold and 2, 9 fold in **B.** U266 - HS-5 co-culture (p <0.05), 2, 3-fold and 2, 5-fold in **C.** primary MM cells – HS-5 co-culture (p < 0.05) after 48 and 72 hours respectively. Mean of Ct values were normalized to RNU44 housekeeping snoRNA or GAPDH and expressed as 2-DDCt value calculated using the comparative cross threshold method. Values represent mean ± SD of three independent experiments. * indicates p < 0.05.

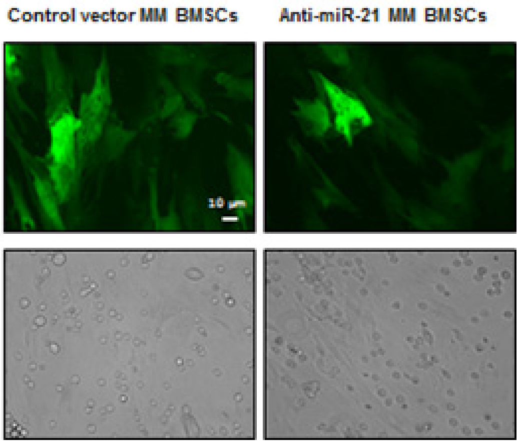

Supplementary Figure S4: Fluorescent microscopy images of a representative MM BMSCs co-culture at 20x magnification. Upper panels. Fluorescent microscopy analysis of transduction efficiency evaluated as percentage of GFP positive MM BMSCs. **Lower panels.** Phase contrast microscopy image of anti-miR-21 and control vector MM BMSCs.

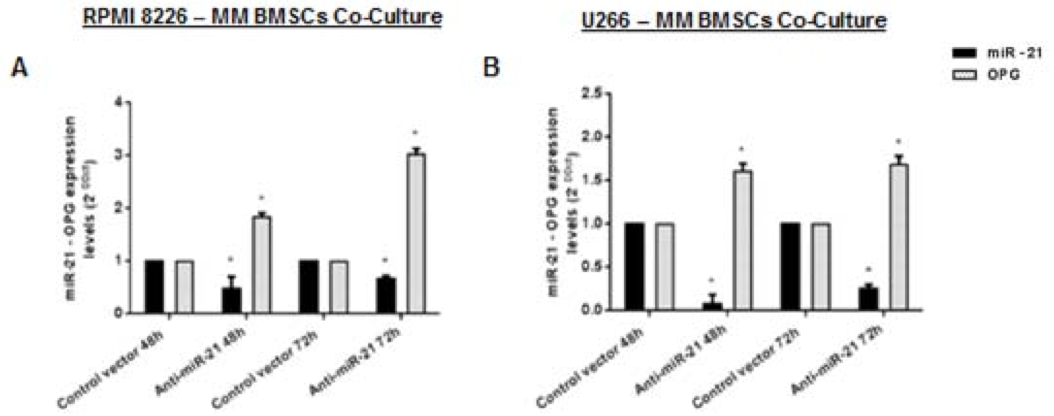

Supplementary Figure S5: Constitutive miR-21 inhibition induces an increased OPG mRNA expression level in MM BMSCs. A and B. Quantitative RT-PCR analysis of miR-21 and OPG expression in primary patient BMSCs transduced with a lentiviral vector carrying miR-21 inhibitory sequences (anti-miR-21 MM BMSCs) or GFP reporter gene (control vector MM BMSCs) adherent to (A) RPMI 8226 and (B) U266. OPG mRNA levels increased by 1, 8-fold and 3-fold in RPMI 8226 – MM BMSCs co-culture ($p < 0.05$), 1, 6-fold and 1, 7-fold in U266 – MM BMSCs co-culture ($p < 0.05$). Mean of Ct values were normalized to RNU44 housekeeping snoRNA or GAPDH and expressed as 2-DDCt value calculated using the comparative cross threshold method. Values represent mean ± SD of three independent experiments. * indicates $p < 0.05$.

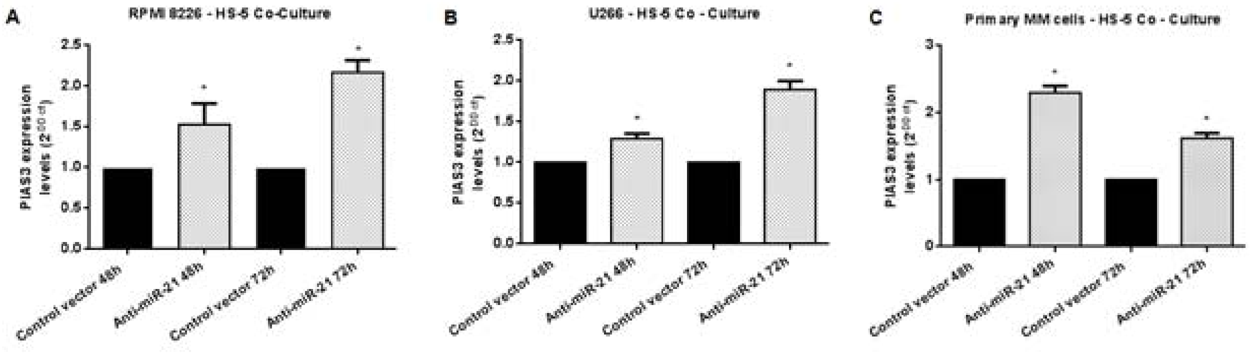

Supplementary Figure S6: Constitutive miR-21 inhibition induces an increased PIAS3 mRNA expression level. A, B and C. Quantitative RT-PCR analysis of PIAS3 in anti-miR-21 or control vector HS-5 adherent to (**A**) RPMI 8226, (**B**) U266 and (**C**) primary MM cells. Mean of Ct values were normalized to GAPDH and expressed as 2-DDCt value calculated using the comparative cross threshold method. Values represent mean ± SD of three independent experiments. *indicates $p < 0.05$.

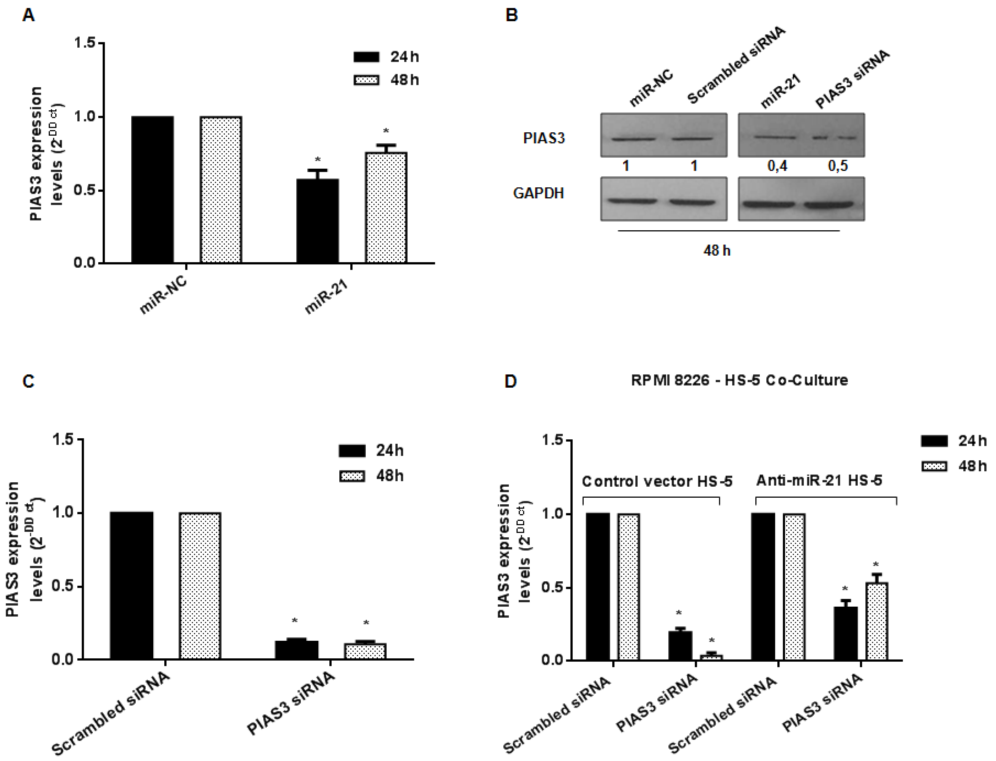

Supplementary Figure S7: miR-21- and siRNA- mediated PIAS3 downmodulation. **A.** Quantitative RT-PCR analysis of PIAS3 in HS-5 transfected with synthetic miR-21 or miRNA scrambled (miR-NC). Analysis was performed 24 and 48 h from transfection. Mean of Ct values were normalized to GAPDH and expressed as 2-DDCt value calculated using the comparative cross threshold method. Values represent mean ± SD of three independent experiments. **B.** Immunoblot detection of PIAS3 in HS-5 transfected with specific PIAS3 or scrambled stealth siRNAs and in HS-5 transfected with synthetic miR-21 or miRNA scrambled (miR-NC) 48 h from transfection. GAPDH was used as loading control. **C.** Quantitative RT-PCR analysis of PIAS3 in HS-5 transfected with specific PIAS3 or scrambled stealth siRNAs. Analysis was performed 24 and 48 h from transfection. Mean of Ct values were normalized to GAPDH and expressed as 2-DDCt value calculated using the comparative cross threshold method. Values represent mean ± SD of three independent experiments. **D.** Quantitative RT-PCR analysis of PIAS3 in anti-miR-21 and control vector HS-5 transfected with specific PIAS3 or scrambled stealth siRNAs and cultured for 24 and 48 h with RPMI-8226. Mean of Ct values were normalized to GAPDH and expressed as 2-DDCt value calculated using the comparative cross threshold method. Values represent mean ± SD of three independent experiments. *indicates $p < 0.05$.
